# A new method of measuring the thumb pronation and palmar abduction angles during opposition movement using a three-axis gyroscope

**DOI:** 10.1186/s13018-018-0999-3

**Published:** 2018-11-16

**Authors:** Tomoyuki Kuroiwa, Koji Fujita, Akimoto Nimura, Takashi Miyamoto, Toru Sasaki, Atsushi Okawa

**Affiliations:** 1 Department of Orthopaedic and Spinal Surgery, Graduate School of Medical and Dental Sciences, Tokyo Medical and Dental University, 1-4-5, Yushima, Bunkyo-ku, Tokyo, 113-8519 Japan; 2 Department of Functional Joint Anatomy, Graduate School of Medical and Dental Sciences, Tokyo Medical and Dental University, 1-4-5, Yushima, Bunkyo-ku, Tokyo, 113-8519 Japan

**Keywords:** Gyroscope, Motion analysis, Thumb opposition, Thumb pronation, Carpal tunnel syndrome

## Abstract

**Background:**

Thumb opposition is vital for hand function and involves pronation and palmar abduction. The improvement of pronation is often used as one of the evaluation items of the opponensplasty method for severe carpal tunnel syndrome. However, most of the studies used substitution evaluation methods for measurement of the pronation angle. Thus, there is still no appropriate method for measuring thumb pronation angle accurately in carpal tunnel syndrome patients.

In recent reports, a wearable gyroscope was used to evaluate upper extremity motions and it can be possibly used for accurate measurement of the thumb pronation angle along the three-dimensionally moving bone axis.

Thus, we investigated the reliability of measuring thumb pronation using a gyroscope and evaluated whether this method can be used to detect opposition impairment.

**Methods:**

The participants were volunteers with unaffected upper limbs (32 hands) and patients with carpal tunnel syndrome (27 hands). The pronation and palmar abduction angles during opposition movements were measured using a three-axis gyroscope that included a three-axis accelerometer. The gyroscope was fixed onto the first metacarpal bone and the thumb phalanx.

**Results:**

The pronation and palmar abduction angles of the metacarpal bone and the palmar abduction angles of the phalanx significantly decreased in the carpal tunnel syndrome group. The pronation angle of the metacarpal bone during opposition movement peaked later than the palmar abduction angle in all hands.

**Conclusions:**

We were able to measure the thumb pronation and palmar abduction angles using the three-axis gyroscope, and this tool was able to detect impairments of thumb opposition due to carpal tunnel syndrome. This could be a tool for measuring thumb and finger angles and for detecting impairments caused by various diseases.

## Background

The thumb is vitally important for hand function and accounts for 40–50% of hand function [1–5]. The most significant factor contributor to the hand’s function is opposition [6, 7]. Opposition movement plays a key role in hand motions such as pulp pinch, grip, and grasp [2, 8–10]. Thumb opposition movement includes two elements, namely, pronation and palmar abduction [1, 11, 12]. Of these, pronation is essential for grasp and pulp pinch [13]. Carpal tunnel syndrome (CTS) is a disease that causes weakness of the thumb’s muscle because of thenar atrophy, resulting in opposition impairment [11, 14–16]. Hence, the extent of improvement in pronation was often used as one of the evaluation items of the opponensplasty method which is aimed to regain the opposition function equivalent to that of healthy subjects [13, 17–21]. Nevertheless, in these reports, nail tip angle, spatial angle, or Kapandji score were used in the substitution evaluation method for the measurement of the pronation angle. Indeed, these methods can be adapted longitudinal evaluation of a single patient; however, they have a number of shortcomings. The first two are not three-dimensional evaluation methods, and the last one is not an accurate quantitative evaluation method but is a numerical categorized method; therefore, even if thenar atrophy increases because of increasing severity, it does not reflect clearly in the numerical value [22]. Thus, despite the decrease in the pronation of the thumb in CTS patients in actual clinical practice, the lack of an appropriate assessment method to evaluate the patients’ function remains.

Various motion analyses of the lower extremities using a gyroscope and accelerometer have been reported [23–26]. In some recent reports, a gyroscope was used to evaluate upper extremity motions in patients with neuromuscular disorders, such as Parkinson’s disease [27, 28] or Duchenne muscular dystrophy [29]. A gyroscope is small, wearable, and easy to handle [30, 31]; hence, we hypothesized that we could measure the thumb pronation angle along the three-dimensionally moving bone axis using a gyroscope.

Therefore, we devised a method for measuring the angles of thumb pronation using a gyroscope and measured the angles in volunteers and patients with CTS during opposition movements. The purposes of this study were to investigate the reliability of measuring thumb pronation using a gyroscope and to evaluate whether this method can be used to detect opposition impairments.

## Methods

This comparative study to investigate the validity of measuring thumb pronation using a new sensor was approved by the institutional review board of our institution, and all participants provided written informed consent.

### Participants

We recruited 16 patients with CTS and thenar atrophy before surgery (CTS group, 27 hands) and 16 healthy volunteers (control group, 32 hands) between June 2017 and June 2018. Upon recruitment, we obtained information from the patients regarding their chief complaint and the trauma history of their hands. We performed medical interview and physical examination such as CTS induction tests and took X-ray images of the hands of all patients.

As the CTS group, patients were included if they were primarily diagnosed with CTS and planned to undergo carpal tunnel release. The diagnostic criteria for primary CTS included finger numbness; the physical findings of CTS, such as Tinel’s sign or positive results on a compression test and the Phalen test; and an abnormal nerve conduction velocity (NCV) value, based on Padua’s classification [32]. We excluded patients with a history of hand surgery or injury, recurrence after carpal tunnel release, positive physical and imaging findings indicative of first carpometacarpal (CM) or thumb metacarpophalangeal (MP) osteoarthritis that could affect the motion of thumb, suspicion of cervical spine disease, or positive magnetic resonance imaging findings of compression because of a space-occupying lesion.

As the control group, volunteers were included if they had undergone total hip arthroplasty in our hospital and if their age and sex matched those of patients in the CTS group. We excluded patients from the control group if they had a history of wrist, hand, or finger surgery or injury; thumb pain; finger numbness; positive physical findings of CTS; or positive imaging findings of osteoarthritis of the first CM or thumb MP. The reason for recruiting patients who underwent total hip arthroplasty for the control group was that these patients underwent routine X-ray of the hand preoperatively to assess the effect on T cane use; therefore, additional radiation exposure was unnecessary.

### Physical examination and NCV testing

To diagnose CTS and evaluate the extent of impairment of thumb opposition, we obtained the following data before this study was performed. All physical findings were obtained through a physical examination by experienced hand surgeons. The degree of atrophy was evaluated in four stages with a visual inspection. The scores of a manual muscle test were evaluated using the Medical Research Council’s Muscle Scale [33]. Experienced neurologists performed all NCV tests and evaluated these data.

### Apparatus

**Fig. 1:**
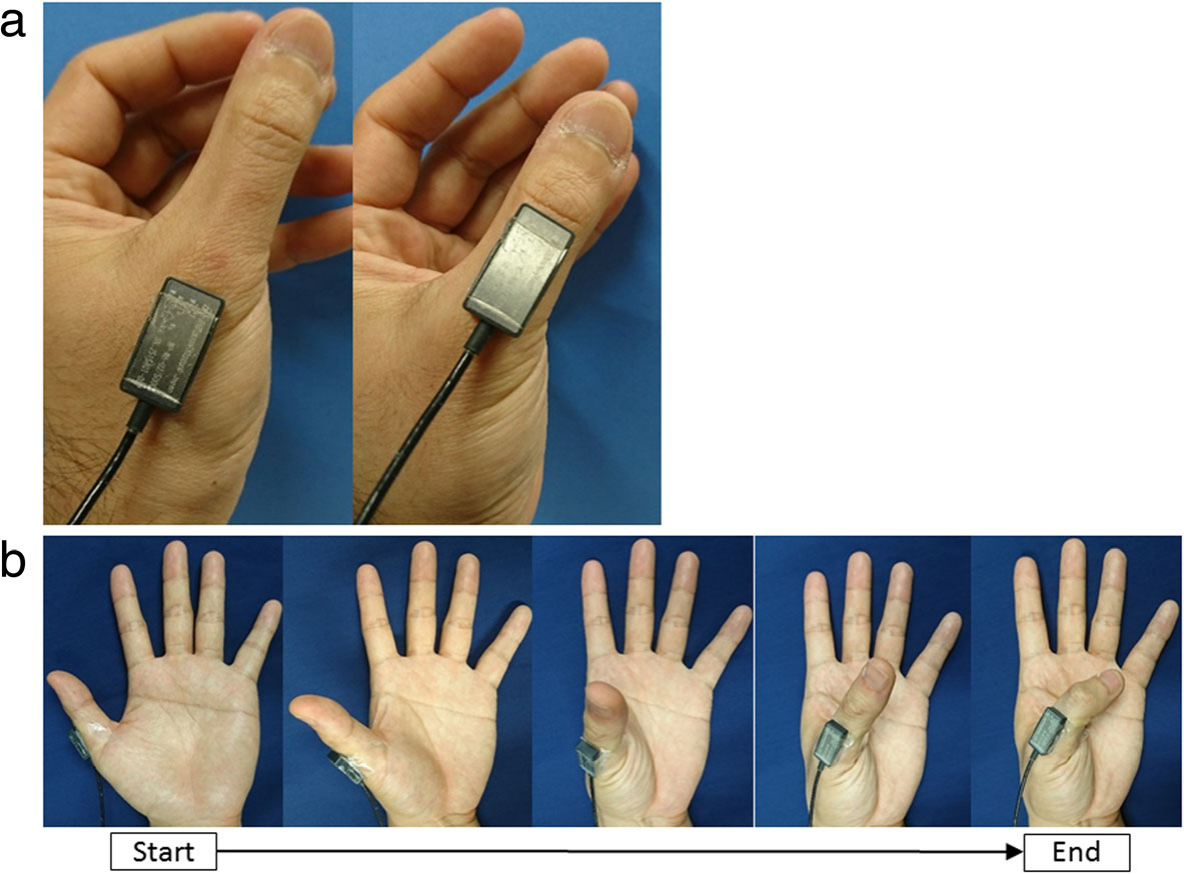
The fixed position of the sensor and the opposition movement during measurement. **a** Left: the sensor on the metacarpal bone; right: on the phalanx along the bone axis. **b** Starting from abduction through full palmar abduction to flexion

The thumb’s angular velocities and accelerations were measured using a three-axis gyroscope with a three-axis accelerometer (MP-M6-02/500C, angular velocity range ± 500°/s; sampling rate 70 Hz, acceleration range ± 20 m/s^2^; sampling rate 200 Hz, size 12 mm in width; 23 mm in depth; 5 mm in height, weight 3 g, MicroStone, Nagano, Japan) during the participants’ opposition movements. The gyroscope was fixed with tape at the dorsal side of the first metacarpal bone or on the middle of the thumb phalanx along the bone axis (Fig. 1a). The gyroscope was connected to a data logger (MVP-RF8, size 45 mm in width; 45 mm in depth; 18.5 mm in height, weight 60 g, MicroStone). The accuracy of this data acquisition method was confirmed in a previous study using the same type of sensor [34]. The gyroscope was calibrated statically against gravity before the measurements were made. The thumb’s angular velocities and accelerations were sampled at 200 Hz, and these signals were synchronized. After analog-to-digital transformation (10-bit resolution), the signals were collected in the logger and immediately transferred to a laptop PC (HP ProBook 450 G2, Hewlett-Packard, Boeblingen, Germany) via a Bluetooth Personal Area Network. It was possible to simultaneously measure the inclination angle of the horizontal plane and the rotation angles of the three axes, and these were calculated with data processing. The working range of the gyroscope to the PC was approximately 80 cm. Signals were processed using commercially available software (MVP-DA2-S, MicroStone).

### Measurement

Participants were instructed to oppose their thumb by drawing as large a semicircle as possible and to move their thumb five times in 45 s. The angles of rotation and inclination of each bone axis were measured during five continuous reciprocating opposition movements, which were from the position of full radial abduction through full palmar abduction to full flexion of the MP and interphalangeal joints (Fig. 1b). These angles were first measured on the thumb phalanx and then on the metacarpal bone. During measurement, the examiner maintained the participant’s wrist and MP joint of fingers in a stationary position.

**Fig. 2:**
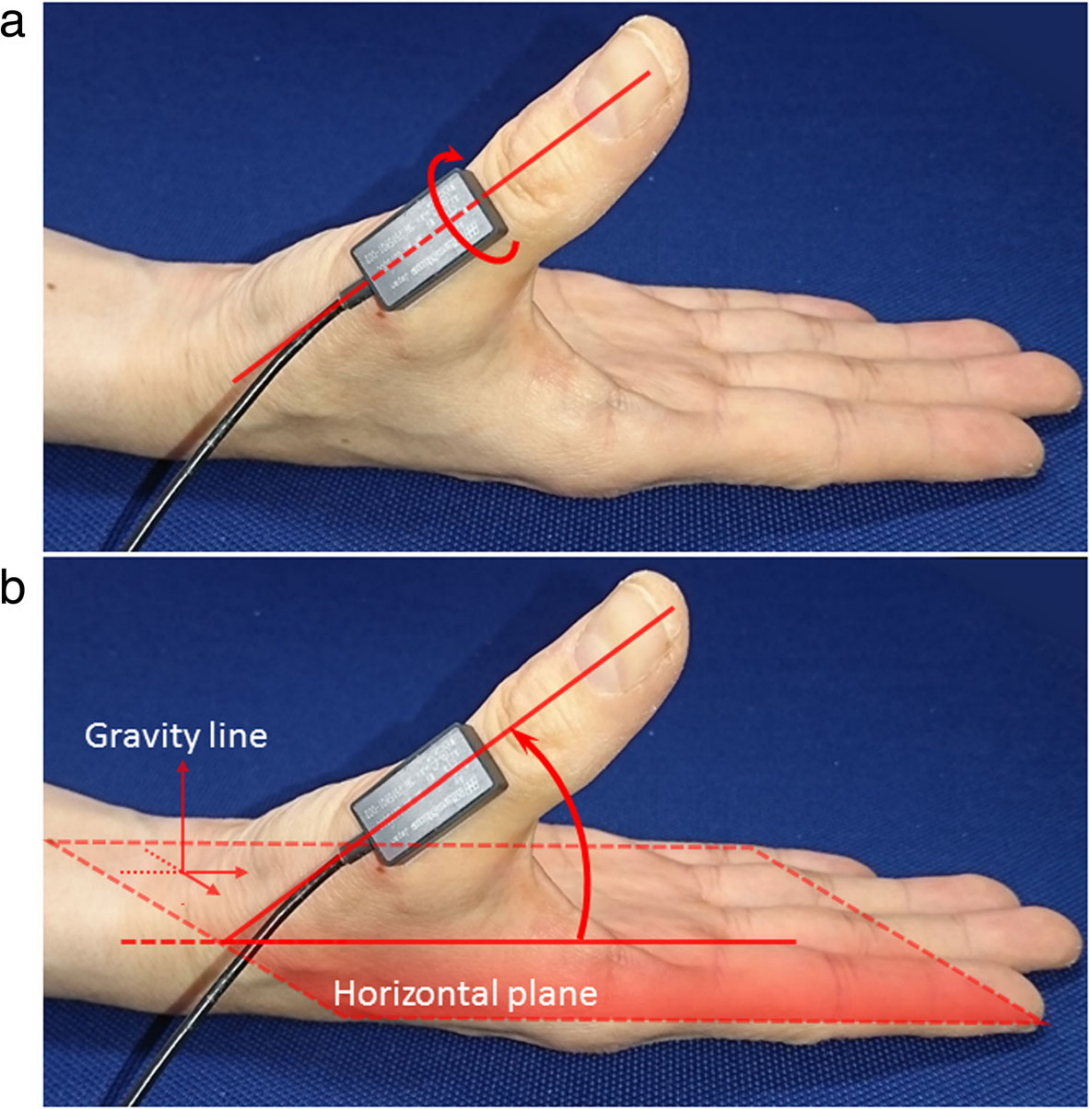
The measured angle during opposition movement. **a** Pronation angle: the rotation angle around the longitudinal axis of the sensor. **b** Palmar abduction angle: the inclination angle of the longitudinal axis of the sensor to the horizontal plane

**Fig. 3:**
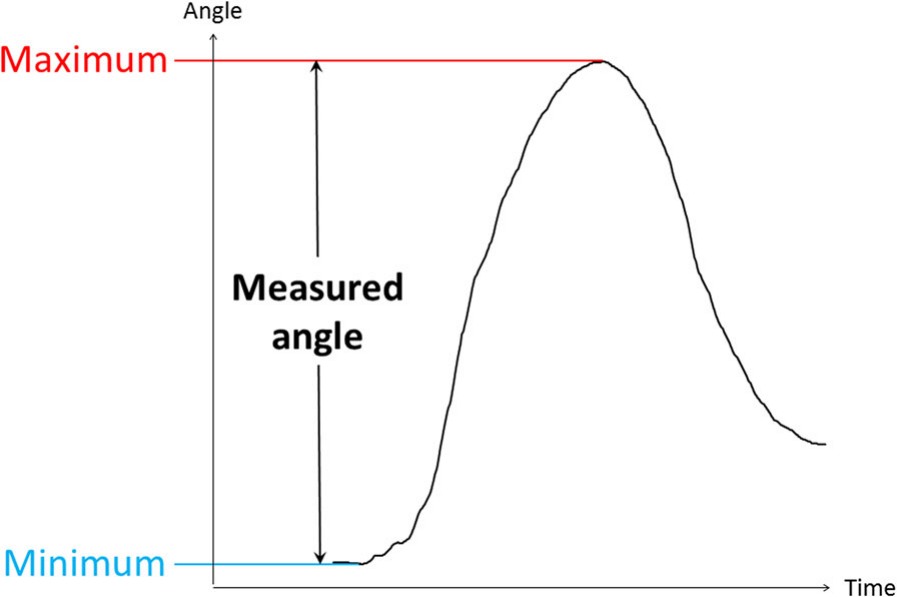
The measured angle. Subtracting the minimum value from the maximum value of the obtained graph

We regarded the rotation angle as pronation/supination and the inclination angle as palmar abduction (Fig. 2). Angle data were evaluated using a system in which the direction of pronation and palmar abduction was considered positive. The difference between the maximum and minimum values was calculated for one opposition movement (Fig. 3). After excluding the first and last opposition movements from the five repeated measurements, we considered the average of these values as the range of motion during one opposition movement. Moreover, we evaluated which peak (pronation or palmar abduction) occurred earlier during one opposition movement.

### Statistical analysis

Data regarding age and the motion angle are presented as the median with an interquartile range. The Mann-Whitney *U* test was used to compare differences. A power analysis was performed on the basis of the pronation angles. To evaluate the intra-tester reliability, the standard deviation of three measurements was calculated for all trials, and the average value was calculated. Using the value, the coefficient of variation (CV) was also calculated. We performed all statistical analyses using EZR (EasyR, version 1.36). *P* <  0.05 was considered statistically significant. We estimated that a sample size of 16 participants per group would be required to achieve 80% power to detect a 10° difference in the aggregated angle for the two groups, assuming an overall standard deviation of 10°, similar to what was observed in a previous study [35].

## Results

### Patient characteristics

The median age was 67.5 (62–76.5) and 68 (54.8–75.5) years in the control and CTS group, respectively. All the participants were female. The physical findings and NCV tests in the CTS group are shown in Table 1. The physical examination showed moderate or severe thenar atrophy in 15 hands, and muscle strength less than good in 13 hands. In nearly all of the patients, CTS was classified as moderate or worse as per Padua’s classification, and only one had mild CTS.

**Table 1:** Data of physical findings and NCV tests in the CTS group

	CTS (*n* = 27)
Thenar atrophy	
Absent	12
Mild	0
Moderate	8
Severe	7
Opposition MMT	
5 (normal)	8
4 (good)	6
3 (fair)	10
2 (poor)	2
1 (trace)	1
0 (zero)	0
Padua’s classification	
Normal	0
Minimal	0
Mild	1
Moderate	16
Severe	6
Extreme	4

*NCV* nerve conduction velocity, *CTS* carpal tunnel syndrome, *MMT* manual muscle test

### Measurement data

The median pronation angle on the first metacarpal was 31° in the control group and 20° in the CTS group. Median pronation angle on the phalanx was 21.5° in the control group and 23° in the CTS group. The median palmar abduction angle on the first metacarpal was 25° in the control group and 18° in the CTS group. The median palmar abduction angle on the phalanx was 55° in the control group and 43° in the CTS group (Table 2). The pronation angle of the metacarpal bone and the palmar abduction angle of the metacarpal bone and phalanx decreased significantly in patients in the CTS group. The average standard deviation of pronation angles of the metacarpal bone and phalanx were 1.3° and 2.4° respectively, and palmar abduction angles of the metacarpal bone and phalanx were 1.3° and 1.9° respectively. The CV of the pronation angles of the metacarpal bone and phalanx were 0.051 and 0.1, and the palmar abduction angles of the metacarpal bone and phalanx were 0.059 and 0.04 respectively (Table 2).

**Table 2:** The range of motion during opposition movement

	Control (*n* = 32)	CTS (*n* = 27)	*P* value	CV
Pronation (°)				
Metacarpal bone	31 (22.8–36.3)	20 (16.5–24.5)	< 0.001	0.051
Phalanx	21.5 (15.3–30)	23 (23.5–33.5)	0.76	0.1
Palmar abduction (°)				
Metacarpal bone	25 (21.8–29.3)	18 (13.5–24)	0.004	0.059
Phalanx	55 (46.5–62.3)	43 (33–49)	< 0.001	0.04

Data are presented as the median (IQR). Statistical significance was determined with the Mann-Whitney *U* test

*CTS* carpal tunnel syndrome, *IQR* interquartile range, *CV* coefficient of variation

**Fig. 4:**
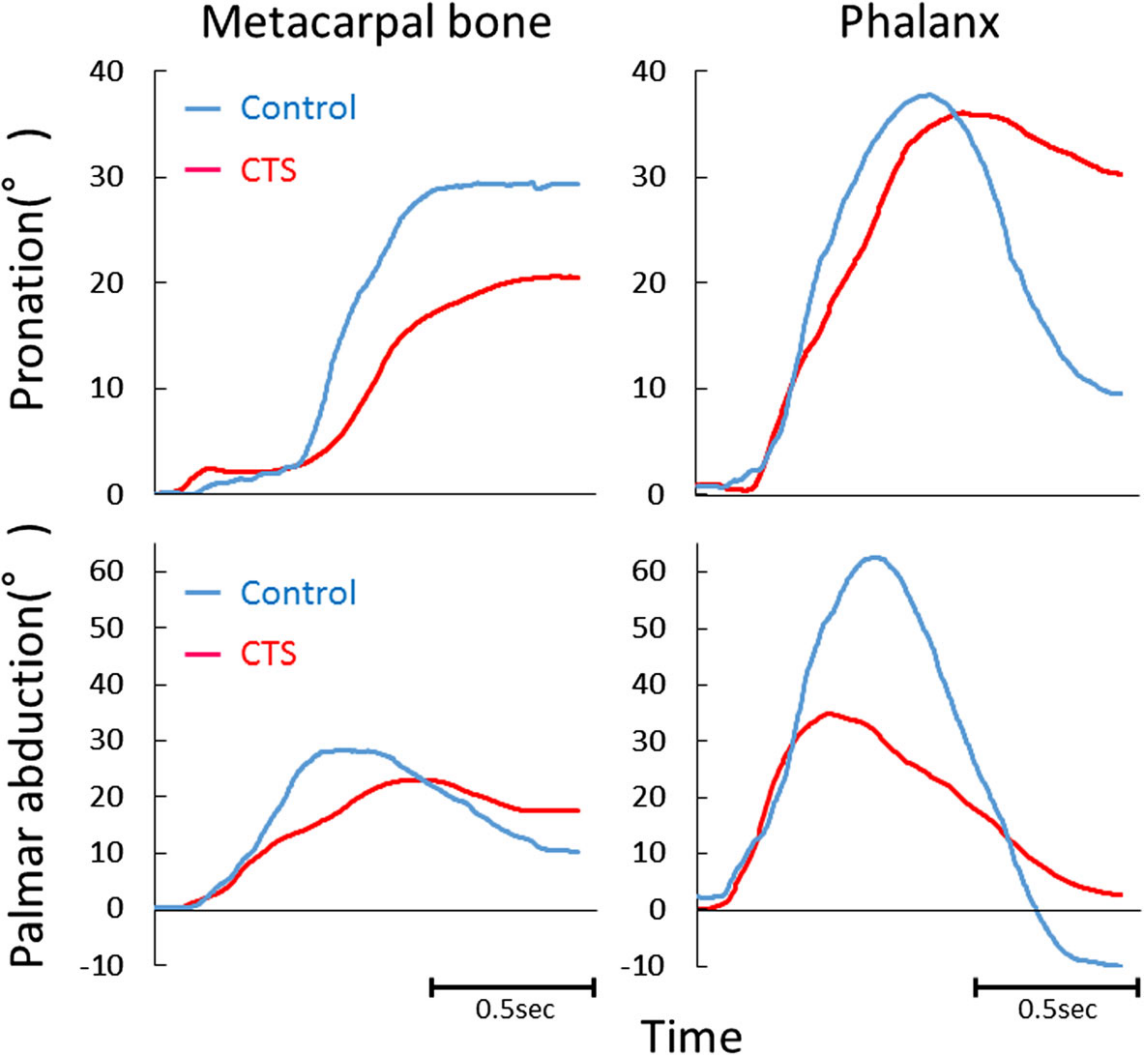
Four representative examples of transition graphs. The measured angles in the CTS (red) and control (blue) groups. Upper: pronation angle; lower: palmar abduction angle; left: metacarpal bone; right: phalanx. CTS: carpal tunnel syndrome

We obtained transition graphs of the measured angles (Fig. 4). Most of the graphs of the pronation angle of phalanx showed a peak near the palmar abduction position during the opposition movement. However, the pronation angle of the metacarpal bone of 19 thumbs in the CTS group and 17 in the control group peaked when the thumb was in the full flexion position. Moreover, in all hands, the pronation angle peaked later than the palmar abduction angle did. The actual measurement time per participant was less than 10 min.

## Discussion

First, we investigated the reliability of measuring the thumb pronation and palmar abduction angles using a small sensor with a three-axis gyroscope and accelerometer. Previous studies have evaluated the pronation angle of the first metacarpal bone using CT in healthy volunteers and yielded various values for the average pronation angle. Cheema et al. and Kimura et al. reported that the pronation angles were 56° and 57° respectively [36, 37]. We considered that the researchers only evaluated the thumb pronation, which was projected into two dimensions using CT imaging; therefore, the thumb pronation angle was overestimated as the palmar abduction angle became closer to 90°. By contrast, Goto et al. and Kawanishi et al. reported that the pronation angle was 14.8° and 22.3° respectively using three-dimensional evaluation method [38, 39]. However, since the former showed the angle from the adduction position to the full flexion position as the pronation angle, it is reasonable that the numerical value becomes lower than ours. Moreover, their report had only one participant. The latter evaluated the differences from the radial abduction position to the full flexion position as the pronation angle using not dynamic CT but the static one. Therefore, it is reasonable that they underestimated the maximum pronation angles of the participants whose thumb pronation angle became maximum slightly later than the palmar abduction position. Moreover, most classical studies showed that the arc of pronation of the first metacarpal bone was less than 30° [40].

Meanwhile, there are many reports of CV measurement of the range of motion of finger and wrist previously. Most of these were about 0.05–0.06 using the gyroscope, optical motion capture, and goniometer [41–43], and almost all the results of our study were similar values.

Hence, we considered that our method to measure the thumb pronation three-dimensionally using a gyroscope is sufficiently reliable.

We believe that measuring thumb pronation angle using a gyroscope has many advantages. Kapandji scores [44] are widely used to evaluate thumb opposition in clinical practice [20, 45–52]. However, it is not an accurate quantitative evaluation method but only a numerical categorized method; moreover, there were reports that almost all healthy subject and even CTS patients with thenar atrophy were able to obtain 9 or 10 points of this score [22, 53]. Although three-dimensional CT was able to measure the angle accurately, it is costly, requires time and effort, and above all, it is very invasive. Optical motion capture systems are dynamic, three-dimensional, and non-invasive but complicated and also require time, effort, and, moreover, a large specialized apparatus. In contrast, a gyroscope is compact, wearable, economical, easy to handle [30, 31], three-dimensional, and non-invasive.

Second, we were able to measure the pronation as well as the palmar abduction angles of the thumb during opposition movements in patients in the control and CTS groups. As expected, there was a significant decrease in the pronation angle of the metacarpal bone and palmar abduction angle of the metacarpal bone and phalanx in patients in the CTS group.

There is only one report of the comparison of thumb pronation angle along the three-dimensionally moving bone axis between healthy subjects and CTS patients using an optical motion capture system [35]. However, the pronation angle in the CTS group decreased, but not significantly. Conversely, our study showed a significant decrease in this angle in patients with CTS. We considered that the discrepancy in these results was because of the difference in the severity of CTS between patients in the previous study and those in ours. In our study, we recruited only CTS patients with thenar atrophy before they underwent surgery, and the disease in almost all of these patients was classified as moderate or worse according to Padua’s classification. By contrast, it is reasonable to assume that the CTS group in the previous study included patients with minimal or mild CTS or patients without a motor disorder. This is because previous reports included (i) only patients with abnormal NCV values, while the threshold was not mentioned and (ii) those with a score of at least 1.5 on the Severity Scale [54]. Furthermore, our study had more than twice the number of cases that this previous study did. These differences in the characteristics of participants may account for the differences in results.

Interestingly, in this study, we also demonstrated a decrease in the angle on metacarpal bone. As CTS causes atrophies of the abductor pollicis brevis muscle and opponens pollicis muscle [55], the result is anatomically feasible. Furthermore, the fact that this method was able to measure the pronation of the metacarpal bone may suggest the applicability of the method to diseases other than CTS, such as osteoarthritis of the CM.

The advancement and importance of our method are anchored on two points. First, by miniaturization of gyroscopes, we succeeded in measuring the thumb pronation angle, which had not been previously measured with a gyroscope. Second, our method allowed the detection of the pronation angle impairment due to CTS easily, non-invasively, and three-dimensionally and represented it numerically.

This study has several limitations. First, it is possible that stretching of the skin while the sensor was applied affected the results. However, this effect should have reduced the measured angle, thereby making it more difficult to obtain any significant outcome. Second, we did not measure the angles of the metacarpal bone and phalanx simultaneously; therefore, we were not able to evaluate the phalanx angle independently. Third, six patients who used canes or walkers were included because the control group consisted of patients with hip osteoarthritis rather than healthy volunteers. Thus, the mechanical effect of a T cane on the first web may have affected the results.

We plan to perform further studies involving simultaneous measurement of the first metacarpal bone and phalanx of the participants and to apply this measurement technique in patients with mild CTS and those with other diseases such as osteoarthritis of the CM. Furthermore, we plan to use this method to evaluate the differences in the pre- and post-opponensplasty pronation angles. In the future, we intend to establish this method as a diagnostic tool for CTS clinically.

## Conclusion

We were able to apply a gyroscope as a new measurement method of pronation and palmar abduction angles of thumb. It was easy, quick, and non-invasive. Furthermore, we were able to demonstrate the significant decrease in the pronation angle of the metacarpal bone and palmar abduction angle of the metacarpal bone and phalanx in patients in the CTS group using the method.

## Abbreviations

CM: Carpometacarpal
CT: Computed tomography
CTS: Carpal tunnel syndrome
CV: Coefficient of variation
MP: Metacarpophalangeal
NCV: Nerve conduction velocity
